# Supplementary material for: Genetic control of dynamic brain network reconfiguration during working memory
Source: PLoS One. 2026 Mar 2;21(3):e0339570. doi: 10.1371/journal.pone.0339570 (PMC12952623; doi:10.1371/journal.pone.0339570)
Supplement: Attachment — Submitted filename: Response to Reviewers (1).docx [file pone.0339570.s002.docx]

**Dear Dr. Tanigaki,**

**Thank you for your time and consideration in reviewing our manuscript. We sincerely appreciate your valuable feedback and guidance throughout the process.**

**We have answered the referee comments and revise the manuscript according to their commands. In the continuation of the text, we answered the referee 1 and 2 and highlighted the changes in the manuscript in blue (for referee 1) and red (for referee 2).**

**Kind regard**,

**Response to Reviewers**

We sincerely thank the reviewers for their careful reading of our manuscript and for their constructive feedback. Below, we address each comment in detail.

**1. Is the manuscript technically sound, and do the data support the conclusions?**

- **Reviewer #1:** Yes
- **Reviewer #2:** Partly

**Response:**
We appreciate the acknowledgment of the technical rigor. Regarding the concerns from Reviewer #2, we would like to clarify that all experiments were conducted with rigor, including the use of appropriate controls, adequate sample sizes, and replication in twin pairs (MZ and DZ). The analyses were performed using established statistical models (e.g., AE twin model for heritability, k-means clustering for dynamic states), and multiple comparison corrections were applied where appropriate. All conclusions drawn in the manuscript are directly supported by the data. We have also clarified in the Methods section how differential phenotypes (2-back minus 0-back) were used to isolate working-memory–specific effects, enhancing interpretability and reducing potential confounds.

**2. Has the statistical analysis been performed appropriately and rigorously?**

- **Reviewer #1:** Yes
- **Reviewer #2:** I Don’t Know

**Response:**
We have conducted thorough statistical analyses consistent with best practices in the field. For static and dynamic connectivity, we applied partial correlations, paired t-tests, and AE twin modeling to quantify heritability. Multiple comparisons were controlled using Bonferroni correction and appropriate FDR procedures. For dynamic connectivity, k-means clustering was used to identify recurring brain states, and temporal metrics (occupancy, dwell time) were rigorously quantified. To enhance clarity, we have now added explicit statements in the Methods section summarizing each statistical approach, assumptions, and the rationale for model selection.

**3. Have the authors made all data underlying the findings in their manuscript fully available?**

- **Reviewer #1:** Yes
- **Reviewer #2:** No

**Response:**
We apologize for any ambiguity. Yes, the data underlying the findings are fully available in compliance with PLOS ONE’s data policy. Please note that the original link to the HCP 1200 Subjects Release dataset has been updated on the official site. The new URL for accessing the HCP 1200 subject dataset is:
<https://humanconnectome.org/study/hcp-young-adult>

**4. Is the manuscript presented in an intelligible fashion and written in standard English?**

- **Reviewer #1:** Yes
- **Reviewer #2:** No

**Response:**
We thank the reviewer for this feedback. The manuscript has been carefully revised for clarity, grammar, and readability. Technical terms have been clarified where needed, and sentence structure has been optimized for better flow. Additionally, figure captions and table legends have been edited to enhance comprehension for readers unfamiliar with specific methods.
We have addressed all concerns regarding technical rigor, statistical methods, and data availability. The manuscript now includes clarifications on:

- Justification for the use of the 2-back minus 0-back differential phenotype.
- Details on statistical tests, corrections for multiple comparisons, and criteria for heritability assessment.
- Explicit description of how dynamic brain states were identified and analyzed.
- Enhanced figure captions and table legends to improve interpretability.

We believe these revisions fully address the reviewers’ concerns and strengthen the manuscript’s clarity and rigor. We sincerely appreciate the reviewers’ time and constructive feedback, which have helped improve the quality and presentation of our work.

**Reviewer #1: This is a nice paper where the authors applied genetic modeling to both static and dynamic connectivity measures and show low heritability (16%) for static connectivity within the Frontoparietal Control Network (FPCN) and Dorsal Attention Network (DAN), but moderate heritability (30%) in the mean dynamic connectivity between the Temporal Gyrus and Frontal Eye Fields, Anterior PFC and Frontal Eye Fields, Lateral PFC and Lateral Intraparietal Area, and Temporal Gyrus and Lateral PFC. They further found that the variance of dynamic connectivity exhibits the highest heritability (78%), particularly in connections between the Dorsal Anterior Cingulate Cortex and Lateral PFC, as well as the Lateral PFC and Posterior Cingulate Cortex.
The authors conclude that the findings highlight that dynamic connectivity variability during working memory tasks is more strongly influenced by genetic factors than static connectivity.**

**Overall, the paper is clearly written, however, the Discussion section seems weak. The authors talk about the SN, DMN, and FPN, and also show that the variance of the dynamic connectivity between SN-FPN and FPN-DMN is very strongly influenced by genetic factors, but do not discuss these in light of the triple network model recently proposed, which posits that dynamic interactions and switching (i.e., variance in authors' current manuscript) between the SN, DMN, and FPN is foundational for many higher order processing in the human brain. The authors should add a separate sub-section in the Discussion section to discuss their findings in the context of the triple network model using the following references:**

**[1] https://www.nature.com/articles/s41467-021-23509-x
[2] https://academic.oup.com/cercor/article/26/5/2140/1754229
[3] https://elifesciences.org/articles/99018
[4] https://academic.oup.com/cercor/article/30/10/5309/5840454
[5] https://www.sciencedirect.com/science/article/pii/S1053811922000568
[6] https://academic.oup.com/cercor/article/32/23/5343/6524032
[7] https://www.sciencedirect.com/science/article/pii/S0010945221003701
[8] https://academic.oup.com/cercor/article/34/7/bhae287/7718277
[9] https://link.springer.com/article/10.1007/s00429-010-0262-0
[10] https://elifesciences.org/articles/76702**

**The authors should also discuss whether the static or the dynamic connectivity is better aligned with the triple network model in the context of genetic influences. This will put the authors' findings in a stronger theoretical grounding.**

**Response:**

We would like to thank you sincerely for your thoughtful and constructive comments, especially regarding the theoretical grounding of our findings in the context of the triple‑network model (SN–FPN–DMN). Your suggestion to explicitly relate our heritability results to this framework has strengthened the manuscript and helped us to provide greater depth and clarity in the Discussion.

In response, we have made the following revisions in the new version of the manuscript:

1. We performed additional analyses using **differential phenotypes (twin difference approach)** to better capture the individual variability in dynamic connectivity. This approach allows us to isolate genetic contributions to connectivity variability while controlling for shared environmental factors.
2. We expanded the Discussion to include a dedicated sub‑section for *Implications for the Triple Network Model of Cognitive Control*, where we analyze and interpret the results of these differential phenotypes in the context of SN–FPN–DMN interactions.
3. The key references you recommended [1–10] have been integrated to situate our empirical findings within the broader literature on network switching, cognitive control, and genetically influenced neural flexibility.

These revisions allow us to highlight that dynamic connectivity variance—particularly in SN–FPN and FPN–DMN interactions—is strongly heritable, reflecting genetic regulation of network flexibility and state switching. Static connectivity and mean dynamic connectivity are discussed in comparison, emphasizing that dynamic variability aligns most closely with the triple-network model in the context of genetic influences.

We believe these updates enhance the theoretical framing and analytical rigor of the manuscript, and we hope that you find the revised version improved accordingly. Thank you again for your time and valuable feedback.

**Reviewer #2: The present study utilized the HCP1200 dataset to investigate the heritability of both static and dynamic functional connectivity (dFC) in twins during an N-back working memory task. The findings indicate low heritability (16%) for static functional connectivity, but a moderate genetic influence (30%) on the mean of dynamic functional connectivity (dFC), and an even higher heritability (78%) for the variance of dFC. This suggests that fluctuations in brain connectivity over time are more genetically driven than static connectivity measures, supporting the notion that network flexibility may serve as a potential endophenotype for cognitive traits.**

**While the study addresses a potential gap in the literature, several major conceptual, methodological, and presentational concerns limit the impact and interpretability of its findings.

The justification for focusing on the working memory task was limited to the statement that "heritability estimates in task-based functional connectivity remain underexplored." This claim seems contradictory to the following citations.**

**Response:**
We appreciate the reviewer’s useful comment. We selected the working memory task because it provides robust activation of large-scale cognitive networks and is one of the most widely used paradigms for studying individual differences in functional connectivity. Our intention was not to generalize across all task domains but to perform a focused analysis of genetic influences during working memory. We now clarify this in the revised manuscript and note that exploring genetic effects across other HCP tasks is an important direction for future work.

**Furthermore, the decision to omit the 0-back condition from the analysis was not justified, especially given the interleaved block design of the N-back task. The 0-back condition serves as an active baseline, controlling for basic visual processing and motor response. Its exclusion raises concerns about whether the reported connectivity patterns reflect working memory-specific processes or more general task-engagement effects.**

**Response:**
We thank the reviewer for this valuable suggestion. We agree that the 0-back condition provides an essential active baseline controlling for perceptual and motor components in the N-back paradigm. In response, we revised our analyses by incorporating the 0-back condition and constructed a differential phenotype (2-back minus 0-back) to isolate the working-memory–specific component of functional connectivity. This contrast reduces variance associated with general task engagement, visual processing, and button-press responses, thereby yielding a cleaner estimate of connectivity changes attributable specifically to working memory load. All heritability analyses (static FC, mean dFC, and variance dFC) were recomputed using this differential phenotype, and the updated results are now reported in the manuscript. We appreciate the reviewer for highlighting this important point, which significantly improved the specificity and interpretability of our findings.

**The study did not explore whether its findings are robust across different cognitive modalities (e.g., face, place, and tool), which can be a compelling extension. This could determine if the high heritability of dFC variance is a general property of working memory or specific to certain modalities.**

**Response:**
We thank the reviewer for this insightful comment. We fully agree that examining whether the observed heritability patterns generalize across different cognitive modalities (e.g., face, place, or tool stimuli) represents an important direction for future research. Our study focused specifically on the working-memory component of the N-back task, and our updated differential analysis (2-back – 0-back) was designed to isolate load-dependent connectivity rather than modality-specific processing. As noted in the Discussion, extending the current framework to modality-dependent contrasts would allow testing whether the high heritability observed in dFC variance reflects a general neural property of working memory or is specific to certain categories of stimuli. We consider this an exciting avenue for future work and have now highlighted this point in the revised manuscript.

**“only 5% of the connections showed significant differences between MZ and DZ twins. This suggests that static connectivity alone has limited heritability and may not be a strong candidate for an endophenotype”. The term "strong endophenotype" remains poorly defined. The field would benefit from the authors providing a clearer benchmark or context for what proportion of heritable connections would constitute a "strong" candidate. Similarly, the descriptor "moderately heritable" for a 30% heritability estimate is subjective. This should be contextualized by comparing it to heritability estimates for other neurological traits or phenotypes in the literature.**

**Response:**
We appreciate the reviewer’s thoughtful feedback regarding the terminology surrounding “strong endophenotype” and the interpretation of heritability magnitudes. We agree that the field would benefit from clearer benchmarks, and we have revised the manuscript accordingly. Specifically, rather than relying on subjective descriptors such as “strong” or “moderate,” we now interpret the heritability estimates in the context of established ranges from the broader neurogenetics literature. For example, structural measures such as cortical thickness and surface area typically exhibit high heritability (40–80%), whereas task-based functional measures often yield lower estimates (10–30%). In this context, a 30% heritability estimate for the mean of dFC reflects the upper bound of what is commonly observed for functional traits, whereas the high heritability of dFC variance is exceptional and consistent with what would be considered a strong endophenotypic signature. These clarifications have been added to the revised manuscript to provide a more rigorous and well-supported interpretation.

**The study identified two distinct dynamic states but provided no further characterization of their functional significance. The analysis would be strengthened by investigating what cognitive processes or neural networks differentiate State 1 from State 2 (e.g., by examining network power or spatial topology) to move beyond a purely statistical description.**

**Response:**
We appreciate the reviewer’s insightful comment and agree that additional characterization of the two dynamic connectivity states strengthens the interpretability of our findings. In the revised manuscript, we have incorporated a network-level analysis of state-specific connectivity topology, based on the reviewer’s recommendation and consistent with prior literature on large-scale brain network dynamics.

Using a Glasser-to-Yeo mapping (7-network parcellation), we computed the mean within-network and between-network connectivity for each state.

We now report a detailed network-level analysis using the seven Yeo canonical systems. This approach summarizes complex connectome-level matrices into interpretable network-based measures of within- and between-network connectivity.

The text has been expanded to explicitly describe the nature of the two dynamic states (segregated/transient vs. integrated/dominant), supported by the new statistical results:

- Stronger within-network coherence in Visual (*p* = 0.043) and trend-level Limbic (*p* = 0.05) systems in State 1,
- Stable between-network connectivity across states,
- Markedly different occupancy and dwell times (State 2 dominant and more persistent).

These additions greatly improve interpretability and directly address the reviewer's request for more accessible visualization of the findings.

We appreciate the reviewer’s insight, which has led to a clearer and more informative presentation of the results.

**The presentation of findings in Figures 2 and 3 lacks clarity. The connectome-wide maps are complex and difficult to interpret. To improve comprehension, these figures can be supplemented with focused illustrations that highlight key working memory regions, making the spatial distribution of heritable connections more accessible.**

**Response:**

We thank the reviewer for this valuable comment and agree that connectome-wide visualizations can be visually dense and challenging to interpret. In response, we have **substantially improved Figures 2**  in the revised manuscript by adding neuroscience-interpretable visualizations that highlight the regions most relevant to working memory.

**Minor issues:**

**Figure 4 was missing error bars, which are essential for assessing the variance and reliability of the presented heritability estimates.**

Thank you for pointing this out. Error bars have now been added to Figure 4 to reflect the standard error of the mean, allowing readers to assess the variance.

**The manuscript frequently used the phrase "We should" which was confusing and gave the impression of a proposed future analysis rather than a completed one. Also, the reporting should be consistently in the past tense for actions that were performed.**

We acknowledge that the phrase “We should” could be confusing. The text has been revised throughout to consistently use past tense for all completed analyses, ensuring clarity that these were performed and not proposed future actions.

**Ambiguous terms like "many" should be replaced with specific quantities or percentages (e.g., “According to the heritability analysis using the AE model (Figure 2a) genetic factors influenced many connections across the brain”). Similarly, “We calculate intraclass correlations for twin data and figured out that, ACE model is better describe our data than ADE model. ” The detailed data should be reported.**

Ambiguous terms have been replaced with precise quantities or percentages.

**In results, no need to introduce ACE model again, which was already introduced in methods.**
eferences to the ACE model in the Results section have been removed, as it was already described in the Methods. This reduces redundancy and improves readability.

**The authors should report specific p values instead of < 0.05**

All statistical results previously reported as “p < 0.05” have now been replaced with exact p-values where available, in line with best practices for transparency and reproducibility.

**The link to the open data does not seem to work properly.**

The original link to the HCP dataset has changed. We have updated the manuscript with the correct link: <https://humanconnectome.org/study/hcp-young-adult>
